# A Probiotic-Based Sanitation System for the Reduction of Healthcare Associated Infections and Antimicrobial Resistances: A Budget Impact Analysis

**DOI:** 10.3390/pathogens9060502

**Published:** 2020-06-23

**Authors:** Rosanna Tarricone, Carla Rognoni, Luca Arnoldo, Sante Mazzacane, Elisabetta Caselli

**Affiliations:** 1 Centre for Research on Health and Social Care Management (CERGAS), SDA Bocconi School of Management, 20136 Milano, Italy; rosanna.tarricone@unibocconi.it; 2 Department of Social and Political Sciences, Bocconi University, 20136 Milano, Italy; 3 Department of Medicine, University of Udine, 33100 Udine, Italy; luca.arnoldo@uniud.it; 4 CIAS Interdepartmental Research Centre, Department of Architecture, Department of Chemical and Pharmaceutical Sciences, University of Ferrara, 44122 Ferrara, Italy; sante.mazzacane@unife.it (S.M.); elisabetta.caselli@unife.it (E.C.); 5 Section of Microbiology, Department of Chemical & Pharmaceutical Sciences, University of Ferrara, 44121 Ferrara, Italy

**Keywords:** hospital associated infections, antimicrobial resistance, antibiotic consumption, probiotic-based sanitation system, sanitation, budget impact analysis, costs

## Abstract

Healthcare associated infections (HAIs) and antibiotic resistance have high social and economic burdens. Healthcare environments play an important role in the transmission of HAIs. The Probiotic Cleaning Hygiene System (PCHS) has been shown to decrease hospital surface pathogens up to 90% vs. conventional chemical cleaning (CCC). This study compares PCHS to CCC as to reduction of HAIs and their severity, related antibiotic resistances, and costs. Incidence rates of HAIs/antibiotic resistances were estimated from a previously conducted multicenter pre-post (6 months CCC + 6 months PCHS) intervention study, after applying the propensity score matching technique. A budget impact analysis compared the current scenario of use of CCC with future scenarios considering increasing utilization of PCHS, from 5% to 50% in the next five years, from a hospital perspective in Italy. The cumulative incidence of HAI was 4.6% and 2.4% (*p* < 0.0001) for CCC (N = 4160) and PCHS (N = 4160) (OR = 0.47, CI 95% 0.37–0.60), with severe HAIs of 1.57% vs. 1% and antibiotic resistances of 1.13% vs. 0.53%, respectively. Increased use of PCHS over CCC in Italian internal medicine/geriatrics and neurology departments in the next 5 years is expected to avert at least about 31,000 HAIs and 8500 antibiotic resistances, and save at least 14 million euros, of which 11.6 for the treatment of resistant HAIs. Innovative, environmentally sustainable sanitation systems, like PCHS, might substantially reduce antibiotic resistance and increase protection of health worldwide.

## 1. Introduction

In times of COVID-19, there is no better moment to talk of infectious diseases and antimicrobial resistance (AMR). AMR is a cause of serious concern for all healthcare organizations regarding clinical, social and economic costs, so much to be considered a real health emergency of the millennium. AMR happens when microorganisms, upon exposure to antimicrobial drugs, become resistant to them. Microorganisms that develop AMR are sometimes referred to as “superbugs”. As a result, the medicines become ineffective and infections persist in the body, increasing the risk of spread to others and the risk of death [1].

The Organisation for Economic Co-operation and Development (OECD) estimated that around 2.4 million people could die in Europe, North America and Australia between 2015 and 2050 due to superbug infections, and that around 1.75 million disability-adjusted life years (DALYs) will be lost unless more is done to stem antibiotic resistance [2]. However, there are large differences between countries, with southern European ones carrying the heaviest burden of AMR. Only in Italy, between 2015 and 2050, the OECD’s model estimates 500,000 deaths and 311,000 DALYs will be lost due to AMR. More recent estimates found that 700,000 deaths each year in the world may be due to bacteria resistant to antibiotics [3]. In the European area, more than 670,000 infections are caused by antibiotic resistance and approximately 33,000 people die as a direct consequence of these types of infections, with over 10,000 deaths in Italy only [4].

The impact of AMR on the healthcare expenditures would be equivalent to USD 1.5 billion per year for EU countries, which means that over the period 2015–2050, the total cost for these health systems would be USD 60 billion. Again, Italy has the highest cost with USD 393 million spent each year by the health system as a result of AMR spread [2].

Excessive and inappropriate use of antibiotics is one of the main factors in the onset of antibiotic resistance in human pathogens. Hospital-isolated microorganisms are more resistant to antibiotics than those isolated in the community. The cause is related to the use, in this context, of a larger quantity of antimicrobial agents, which increases the probability that bacteria develop mechanisms, due to genetic mutations or gene exchange, allowing them to survive despite the presence of antibiotics [5]. An important consequence of antibiotic resistance is represented by difficult-to treat Healthcare Associated Infections (HAI), i.e., infections that a patient contracts during his/her hospital stay, in any care setting, which was absent at the time of admission. There is a strict correlation between AMR and HAIs which represents 75% of antibiotic-resistant infections [6]. HAIs affect 3.2 million patients in Europe each year, leading to about 37,000 deaths as a direct consequence, with increasing prevalent role of multi-drug resistant (MDR) pathogens [7,8]. It is, however, clear that excessive use of antibiotics is not the only cause for AMR. The drivers of AMR are multifactorial and so should be the interventions, including several sectors such as human, animal and environmental health sectors in a “One Health” approach [9]. Simultaneous measures to improve sanitation, infection control and prevention, access to clean water, governance, and public expenditure on health-care need to be implemented to tackle antimicrobial resistance on a global scale [10]. Collignol et al. found that poorer infrastructure, such as poor sanitation was consistently associated with higher levels of AMR and that-for example-we would see *E. coli* resistance levels fall by 18.6% for every unit of improvement in the infrastructure index [10].

Similar results are found in the OECD’s analysis that shows simple measures, such as promoting hand washing and better hygiene in healthcare settings (e.g., hospitals and community), could more than halve the risk of death and decrease the health burden of AMR [2]. The “hospital-based” package alone (including improved hand hygiene, stewardship programs and enhanced environmental hygiene) would reduce the burden of disease from AMR by 85%, produce savings of USD 4.1 per capita per year, avert around 1.3 million DALYs and 55,000 life years saved across the 33 countries included.

In current times, the role of cleaning in hospital settings for managing HAIs and, more in general, for reducing infectious transmission, is of paramount importance. Traditional cleaning based on chemical substances are notoriously of limited efficiency in decontamination [11] as it fails to prevent recontamination [12]; furthermore, chemical disinfection has a high environmental impact and can contribute to the selection of resistant pathogens [13,14].

New approaches have been proposed, including disinfectants, steam, automated dispersal systems, and antimicrobial surfaces [13], but there are a few studies that focus on the impact of enhanced or alternative cleaning practices in the routine situation [15]. An intervention enhancing environmental hygiene entails any of the following three actions: (1) disinfectant substitution, i.e., a change from detergent to disinfectant, or to a different disinfectant assumed to have higher effectiveness against certain pathogens; (2) no-touch cleaning, i.e., use of an automated cleaning device, emitting hydrogen peroxide vapour or ultraviolet radiation, to disinfect rooms after routine cleaning; and (3) improving effectiveness of cleaning, i.e., additional cleaning time through the employment of new staff; audit, monitoring and feedback regarding cleaning practices and thoroughness; staff education as well as novel techniques of applying products, such as using disposable wipes or colour-coded cloths [15]. The average cost of enhanced environmental hygiene interventions was estimated at USD 30.6 per person per year, based on the components of the different types of interventions falling in one of the three categories above, and the effectiveness in reducing the AMR rates ranged between 26% and 49% [2].

Among the new approaches, the Probiotic Cleaning Hygiene System (PCHS^®^), based on ecologically sustainable detergents containing spores of *Bacillus* probiotics, integrates different factors as a specific activation technique for biological competition, the use of specific microfiber materials (combining dusting and washing activities), certified procedures and a microbiological control. These factors guarantee standards including low microbial load and stability over time. Sanitization operations are performed in accordance with the PCHS^®^ protocols, which involve also a specific training plan for staff based on hygiene culture in order to ensure the effectiveness of both process and results.

Experimental research has shown that the PCHS is able to steadily decrease surface pathogens up to 90% more than conventional disinfectants [16,17] without inducing the selection of drug-resistant strains, as demonstrated by molecular analyses of the entire microbiota resistome [17,18,19]. In a multicenter study, the system showed to reduce the HAI cumulative incidence from a global 4.8% to 2.3% (OR = 0.44, CI 95% 0.35–0.54) (*p* < 0.0001), compared to chemical disinfection. Moreover, the antimicrobial resistance genes harbored by surface microbes decreased up to 99%, and consistently the antimicrobial drug consumption associated with HAI onset showed a global 60.3% decrease, with a 75.4% decrease of the associated costs [19,20].

However, although current evidence on PCHS derives from a very large number of patients (N = 11,461) [20], data analysis was performed by comparing the pre-PCHS and PCHS patient groups without any matching of the patients themselves, thus leading to possible bias in the results [19]. The present study aims at filling this gap, by comparing costs and outcomes of the PCHS method vs. conventional chemical cleaning (CCC) after matching groups of patients and measuring the impact of PCHS in terms of (i) reduction of HAIs, (ii) HAIs outcomes, (iii) antibiotic resistance rates, (iv) economic costs.

## 2. Materials and Methods

### 2.1. Study Design

A pre-post intervention study [20] has been conducted from 1 January 2016 to 30 June 2017 in five Italian public hospitals (plus a control hospital) with the aim to assess HAI incidence. The study developed in two phases: a 6-month pre-intervention investigation phase, in which hospitals maintained conventional chemical cleaning (CCC) procedures based essentially on the use of chlorine-based products, and a 6-month intervention phase (PCHS), which involved the use of the PCHS system. Briefly, CCC consisted of sodium hypochlorite 0.1%, whereas PCHS eco-labeled detergent contained 10^7^ probiotics/mL and was used diluted 1:100 in water. Both CCC and PCHS were applied daily. The departments of internal medicine/geriatrics and neurology were chosen in order to have a sample of homogeneous patients for the estimation of the HAI incidence. A total of 5930 and 5531 patients were enrolled in CCC and PCHS phases, respectively.

In this study, in order to make patients in the CCC and PCHS groups comparable, we used propensity score to best account for the unavoidable selection bias [21,22,23]. More specifically, a one-to-one nearest neighbor propensity score matching (PSM) procedure has been performed in order to identify the two subsets of patients. We constructed a propensity score as the logit function of the probability of being managed with PCHS or conventional cleaning procedures for a patient with specific baseline characteristics. The patients’ characteristics and prognostic factors were selected by consensus among the clinicians involved in the study, with focus on parameters which reported statistically significant differences between the two groups: age (*p* = 0.0001), gender (*p* = 0.003), presence of urinary catheter (*p* = 0.01), self-sufficiency (*p* = 0.009), presence of pressure sores (*p* = 0.00001), presence of mechanical ventilation (*p* = 0.00001), use of antibiotics in the last two weeks (*p* = 0.00001), parenteral nutrition (*p* = 0.00001), presence at admission of multi-drug resistance (MDR) organisms (*p* = 0.008). The reason of hospital admission has been excluded from the set of variables used in the matching process because not deemed relevant for the analysis of HAIs. STATA 14 software and the command psmatch2 [24] have been used to perform the PSM.

### 2.2. Study Outcomes

The outcomes measured in this study are: (i) number of HAIs per 1000 patient days in both CCC and PCHS periods; (ii) HAIs outcomes; (iii) consumption of drugs to treat HAIs and the related costs; (iv) identification of cases of antibiotic-resistance and estimation of their treatment costs. The cases of antibiotic-resistance have been calculated by assuming the occurrence of antibiotic-resistance when a change of therapy is performed, consistently with what assumed in previous studies in the same area [25,26,27,28]. In particular, for each patient, we classified as antibiotic-resistance the cases in which the number of antibiotics administered were higher than the number of HAIs for that patient (e.g., two drugs for the management of one HAI).

As regards the HAIs outcomes, these were evaluated in terms of severity of adverse events according to the Australian Incident Monitoring System (AIMS) [29], which classifies adverse events according to 8 levels of severity from none to severe/fatal consequences.

Since we are aware that a careful risk assessment needs to be carried out to take this biological method into wider use avoiding potential harmful effect, the safety of PCHS use was ascertained both by assessing any potential infectious ability of PCHS *Bacillus* per se in the hospitalized patients, and by analyzing the PCHS *Bacillus* genetic features. Briefly, a microbiological surveillance was implemented in each healthcare center using PCHS, where *Bacillus* spp. are included as ‘alert organisms’ in the search of potential infectious microbes performed by the hospital’s microbiology laboratory.

### 2.3. Healthcare Resource Consumption and Costs

An economic analysis has been conducted to estimate the costs (EUR, 2019) of pharmacological treatments to treat HAIs in the CCC and PCHS groups, with a subgroup analysis performed on cases of antibiotic-resistance. The cost for drug treatment was calculated starting from the daily cost per patient. Each hospital was asked to provide, for antimicrobial drugs (including antibiotics and antifungals), the daily cost per patient with standard dose, distinguishing by different routes of administration. In case of missing data for a hospital, an average cost was calculated based on data provided by the other hospitals for the same drug. Table 1 shows for each drug the daily cost or the minimum and maximum values found in the different hospitals for the different routes of administration. A single value reported for a drug/route of administration means that the cost was the same for the different hospitals or that only one clinical center used it (this latter case is identified by an asterisk).

The total cost per patient was calculated by multiplying the daily cost by the duration of the treatment and summing up the costs of all different treatments administered to him.

As to the cost of the cleaning methods, it has not been estimated since they were equivalent, as emerged from public national procurement tenders awarded to PCHS provider [30]. Appendix A (Table A6) shows the costs of traditional chemical cleaning vs. PCHS in terms of working hours, based on three types of areas and on the same sets of cleaning activities.

Hospital length of stay was also recorded for patients in the CCC and PCHS groups and for those who developed antibiotic-resistance in order to estimate any difference in the two groups.

### 2.4. Budget Impact Analysis

A budget impact model has been developed in order to evaluate the expected changes in the expenditure for the Italian hospitals for the pharmacological treatment of HAIs and related drug-resistance in the hypothesis of the utilization of PCHS vs. the traditional cleaning method.

In Italy, in 2018, the annual hospital discharges related to internal medicine/geriatrics and neurology departments (i.e., the same population considered in our analysis) were about 1,288,000 [31]. Since the PCHS use in Italy is at the moment very limited (less than 5% of hospitals according to data provided by the manufacturer), the baseline scenario of patients’ distribution between the two alternative sanitation systems considered 100% of use of CCC. The introduction of new technologies, as the PCHS, often encounters difficulties for example because organization(s) are inadequately set up for innovation or are not interested/ready for it, or are unable to negotiate a viable business model with partner organizations, or because the intended users of the technology have plausible personal (e.g., diffidence) or professional reasons to resist or reject it [32,33,34]. For these reasons, future scenarios considered modest increasing utilization rates for PCHS of 5%, 10%, 15%, 30% and 50% over conventional cleaning procedures for the following 5-years. The model applies the variations of the market share to the incident cohort of patients (assumed constant in the years).

The costs for current and future scenarios were estimated by multiplying yearly costs of each option by the proportion of the population managed with that option and by the number of patients in the considered population, taking into account a constant cohort of incident patients. As the focus was on the expected budget at each point in time, the financial streams were presented as undiscounted costs [35].

## 3. Results

### 3.1. Target Population, Pharmaceutical Treatments, HAI and Antibiotic-Resistance Rates, HAIs Outcomes

The propensity score matching was performed on 11,461 total patients included in the study [20], varying the caliper radius. For values less or equal than 0.000001, the PSM yielded a sample of 8320 patients (4160 per group) with identical clinical characteristics (100% reduction bias on all the variables). Table 2 shows the populations’ characteristics.

Patients who developed at least one HAI were 291, 191 during the CCC phase and 100 during the PCHS period, for a total number of HAIs of 203 and 106, respectively. The cumulative HAI incidence (i.e., No of patients with HAI/n. enrolled patients) decreased significantly from 4.6% to 2.4% (*p* < 0.0001) moving from CCC to PCHS (OR = 0.47, CI95% 0.37–0.60).

Table 3 compares the different drugs used in the two periods, total treatment durations, mean number of treatment days by patients with HAIs, and mean number of treatment days by patient with HAI treated with the specific drug. The consumption of synthetic broad-spectrum antimicrobials like quinolones and fluoroquinolones (e.g., levofloxacin, ciprofloxacin) was significantly reduced (from 508 to 70 total days of therapy) with the transition from CCC to PCHS. At the same time, there was an increase (from 34 to 104 total days of therapy) in the use of less expensive first-line antibiotics (e.g., amoxicillin/clavulanate) in the PCHS phase, suggesting that the HAIs bacterial etiologic agents were susceptible to such drugs. The mean treatment duration per patient, calculated by dividing the total days of pharmacological treatment by the number of patients with HAI, was 11.88 and 11.04 days, for CCC and PCHS periods, respectively.

During the CCC period, out of 191 patients who developed a HAI, 47 were assumed to develop antibiotic-resistance according to the methodology presented by Thorpe and colleagues [25]. Analogously, in the PCHS period, 22 patients out of 100 who developed a HAI, were estimated to report antibiotic-resistance. Thus, 1.13% and 0.53% patients (as calculated on the total 4160 patients in each period) showed antibiotic-resistance in the conventional cleaning system and PCHS periods, respectively.

Regarding the HAIs outcomes, these were evaluated in terms of severity. For the CCC period 111 (54.6%), HAIs showed from moderate to significant severity; in three cases (1.5%), patients reported a severe outcome (death or severe disability), while the remaining HAIs reported minor or no consequences. For the PCHS period, these figures were 46 (43.4%) and 1 (0.9%), respectively (Fisher test for comparison *p* = 0.486).

The mean hospital length of stay was slightly, but not statistically, different (Wilcoxon test *p* = 0.13) for patients with HAI in the CCC (17.81 days) and PCHS (20.08 days) groups, and for patients who developed antibiotic-resistance in the same groups (26.62 vs. 30.05 days; Wilcoxon test *p* = 0.90).

### 3.2. Budget Impact Analysis

The economic analysis focused on those patients who developed at least one HAI (291). From our analysis it emerged that the cost for the pharmacological treatment of a patient with HAI decreased from EUR 272 in the CCC period to EUR 110 in the PCHS phase, with a cost reduction of 59.8% per patient. Overall, the cost of medications associated to treatments of HAIs was equivalent to EUR 52,004 in the CCC phase and to EUR 10,954 in the PCHS phase, showing that the PCHS system reduces the total costs of drugs by 78.9% (average cost per patient over the total population: EUR 2.63 versus EUR 12.50). Cost saving was due to both (i) the reduction in the number of patients with HAI, and (ii) to the use of less expensive drugs for the management of infections (Table 3).

As to the cost of treating antibiotic-resistance, it was equivalent to EUR 40,419 and EUR 6341 in the CCC and PCHS periods, respectively, highlighting a saving of 84%. The mean cost for the treatment of each single patient resistant to antibiotics was EUR 859.98 and EUR 288.23, respectively, for CCC and PCHS, showing a cost reduction of 66% (average cost per patient over the total population: EUR 1.52 versus EUR 9.72). The use of PCHS led to a decrease of the number of patients with drug-resistant HAIs and, in turn, to a reduction in drug costs. Again, likewise in the case of HAIs, the reduction of drug cost is also due to the use of cheaper drugs for the management of the antibiotic-resistant infection.

If we apply the percentages found in the two groups of patients to the total yearly number of hospital admissions in the departments of internal medicine/geriatrics and neurology (1,288,000) in Italy, the number of HAIs and drug-resistances would be 59,248 and 14,554 for the conventional cleaning approach vs. 30,912 and 6826 for PCHS, respectively. Of these, the cases of severe HAIs would be 930 and 309 for CCC and PCHS, respectively.

**Figure 1:**
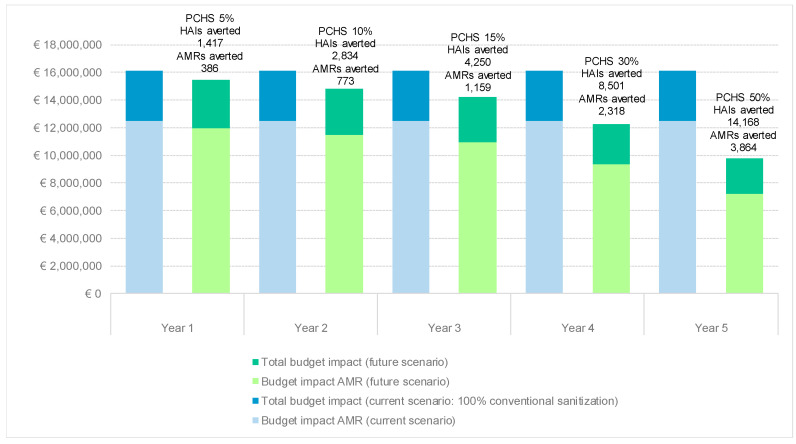
Budget impact comparing current and future scenarios.

Given the clinical outcomes and the same acquisition costs of the different cleaning methods (CCC and PCHS), the progressive switch from chemical cleaning to PCHS would bring considerable cost savings since the first year of PCHS implementation. Namely, compared to the current scenario which considers 100% use of chemical substances, future scenarios considering utilization rates of the PCHS of 5%, 10%, 15%, 30% and 50% over conventional cleaning would avert about 31,000 HAIs and about 8500 AMRs in the next five years. This would allow savings of EUR 635,628, EUR 1,271,256, EUR 1,906,884, EUR 3,813,768, and EUR 6,356,280 for the following 5 years (Figure 1), if we assume new cohorts of patients populating the hospital departments for the pharmacological treatment of HAIs. The total savings over the 5-year time horizon would be EUR 13,983,816. Focusing on the subset of HAIs presenting drug-resistances, under the same assumptions, it would be possible to save about 11.6 million euros (83% of the total savings) in the next five years.

### 3.3. Risk Assessment Analysis

No infection by Bacillus was detected in any patient hosted in the treated settings, including those presented here, in about ten years of PCHS use, and no samples positive for Bacillus presence were detected in over 50,000 clinical specimens [20,36]. Besides, the PCHS-Bacillus species were shown to contain a few chromosomal not-transferable resistance genes, and the gene exchange between them and the surrounding pathogens was assessed in each treated structure in over 600 PCHS-Bacillus isolates from surfaces [17,37]. No acquisition of new resistance genes was detected in any Bacillus sample, supporting their high genetic stability despite the continuous contact with resistant pathogens. Indeed, gene exchange mechanisms are, however, not favoured on hard dry inanimate surfaces.

On another hand, PCHS-Bacillus can persist long in the environment, substantially modifying the pre-existing microbiome by replacing 80% of previous microbes in around one month of treatment. Thus, a ‘rescue’ procedures was set up, able to remove completely the Bacillus spores from the environment, if needed (unpublished results).

For precaution reasons, no wards hosting severe immunodeficiency patients (such as transplanted or leukemia patients) have been included so far in the studies, nevertheless no unexpected adverse effects were observed in elderly patients (the mean age of surveyed patients was >70 years), that are characterized by a constitutive age-related mild immunodeficiency.

However, risk assessment analyses are an integral ongoing part of the PCHS implementation in the treated settings, allowing to monitor each microbiome modulation and any eventual undesired effect.

## 4. Discussion

Latest evidence shows that the levels of drug-resistance and multidrug-resistance of microbial species under surveillance are still very high [38]. Despite the considerable efforts made so far, such as the promotion of appropriate use of antibiotics and interventions for infection control in healthcare facilities, in Europe the composite index of antimicrobial resistance in bacteria from HAIs in acute care hospitals reaches 31.6% [8]. In Italy in 2018, the percentages of resistance to the main classes of antibiotics for the eight pathogens under surveillance (*Staphylococcus aureus*, *Streptococcus pneumoniae*, *Enterococcus faecalis*, *Enterococcus faecium*, *Escherichia coli*, *Klebsiella pneumoniae*, *Pseudomonas aeruginosa* and *Acinetobacter* species) remain higher than the European average, although the trend is declining compared to previous years [6].

A previous study by our group proposed a new approach to the relationship between environment, hygiene and infection prevention and control [20], but matching of patients was lacking, thus leading to possible bias in the results. The present study stem from previous data, elaborated by a propensity score matching to render comparable the groups, aiming to generate potentially helping supporting policies aimed at reducing the development of microbial resistance [14,39].

Following several previous initiatives, in 2017, the Italian Ministry of Health developed the national action plan 2017–2020 (PNCAR) aimed at setting the agenda to fight against HAIs and antibiotic-resistance [40]. In order to monitor progresses in achieving these objectives, some synthetic indicators have been selected in the field of surveillance of antibiotic use, of infections related to care and the percentage of multi-resistant microorganisms. Regarding the antibiotic use in hospitals, the indicators set a reduction in systemic antibiotic use greater than 5% and a reduction of fluroquinolones greater than 10% in 2020 compared to 2016. For the same period, a reduction greater than 10% has been set for the prevalence of MDR in blood isolates. Although the measures included in the PNCAR include hand hygiene and antibiotic stewardship, the possible contribution of hospital environment hygiene is not mentioned. By contrast, our results suggest that an environmental intervention can significantly contribute to the amelioration of HAI and AMR concerns. In fact, PCHS use was associated with a reduction of HAI incidence of 48% and, among total HAIs, the drug-resistant HAIs were reduced by 53% compared with the use of conventional cleaning protocols. Moreover, the PCHS showed a reduction of about 67% of severe HAIs (from 1.57% to 1%). Therefore, the PCHS would allow savings from the Italian hospital perspective of about 14 million euros in 5 years, of which 11.6 related to the pharmacological treatment of drug-resistances. These results by far surpass the objectives of the PNCAR, as with PCHS the use of broad-spectrum antimicrobials like quinolones and fluoroquinolones may be reduced by 86% in terms of treatment days, with drug-resistant HAIs reduced by 53%.

An Italian study [41] conducted on 49 hospitals in 19 regions for a total of 14,784 patients, reported a prevalence of patients with at least one HAI of 6.3%. Another study conducted at European level showed for Italy a HAI incidence of 6% [8]. Since these studies considered all hospital wards, the HAI incidence of 4.6% estimated by our study on patients admitted to internal medicine/geriatrics and neurology departments only, may be considered a conservative estimate.

Had we extended our findings to all Italian hospitalized acute patients (6,502,529 in 2018 [31]), the annual savings for the pharmacological treatment of HAIs would sum up to about 70.6 million euros on a 5-year time horizon. Similarly, had PCHS entirely (100%) replaced CCC in all Italian hospitals, the savings would sum up to 320 million euros in the next 5 years only for pharmacological treatments at no additional costs for the national healthcare service. Again, if we extended our results to the annual number of HAIs reported by the Italian Institute of Public Health (ISS) (450,000 to 700,000 [42]), the annual impact of resistant HAIs in terms of pharmacological treatments would be in the range 95.1–158.4 million euros. This amount is only a part of the wider value of 319 million euros estimated for the direct healthcare cost of antimicrobial resistance in Italy, which considered the additional hospitalization days, i.e., the difference between the mean length of stay with resistant infection and the mean length of stay for the same hospitalizations without infection [43]. In our study, the mean hospital length of stay for patients with HAI was slightly longer for the PCHS group (20.08 days) compared to CCC use (17.81 days), nevertheless, this difference is likely to correlated with specific causes of hospital admissions in the two periods and not with the two cleaning systems as proved by the shorter mean treatment durations per patient for HAIs in the PCHS group vs. the CCC group (11.04 vs. 11.88 days).

The present study has strengths and limitations that need to be discussed. Although randomized controlled trials are to be preferred whenever possible, propensity score matching technique allows to design and analyse real-world studies so to mimic some of the characteristics of randomized controlled trials and provide decision-makers high-quality evidence in less time and at lower costs [44]. In our case this methodology has been applied in order to select two subsets of patients, considering chemical sanitization and PCHS, with overlapping main clinical characteristics. This approach contributed to further confirm the previously published results for which analyses have been performed without patients’ matching [19,20].

Moreover, this study is one of the few published evaluations of both clinical and economic aspects of the management of patients with HAI according to preventive systems and, more specifically, to cleaning approaches. The OECD reported that in Italy a package of actions that includes stewardship programs, better hygiene in healthcare facilities, information campaigns and the use of rapid diagnostic tests could avoid 8800 deaths and save 527 million USD (about 487 million euros) annually [2].

Concerning the study limitations, the measurement of antibiotic-resistance was assumed upon the consumption of antibiotics/HAI, in line with what has been done in previous studies [25]. Nevertheless, more precise estimates could be gathered if a comprehensive collection of antibiograms performed in case of HAIs had been possible in the clinical study [19,20]. The study, which considered the hospital perspective, focused on the consumption of drugs for the management of HAIs and did not consider other cost items like intensive care treatments and the cost for the healthcare personnel, which may have a great impact on the management of HAIs.

Although the use of PCHS saves 3% of cleaners’ time (Appendix A, Table A6), this aspect has been excluded from the present analysis because we preferred to adopt a conservative approach.

In Italy, it has been estimated that over 1000 injury claims for HAIs were filed by public hospitals to receive insurance reimbursements, equivalent to 3.37% of total injury claims in the period 2004–2011; and that the average cost per claim is equivalent to almost EUR 44,000 [45]. In case we applied the cost per claim to the severe HAIs, the use of the PCHS in all Italian internal medicine/geriatrics and neurology departments would generate savings of 27.3 million euros per year.

Another limitation relates to the estimation of the population considered in the budget impact analysis. In the base-case scenario, we referred to the annual number of hospitalizations at national level for the wards considered in the multicentre pre-post study and we applied the estimated mean costs per patient for the treatment of HAI and resistant HAI for PCHS and conventional sanitizing system. In an extended scenario, we applied the study results to the whole Italian hospitals. Although in Italy the management of the risk of hospital infections is deployed through the observance of national guidelines [46], the different centres present heterogeneity in organization plans and sanitizing processes [47]. Moreover, across the nation, heterogeneity may arise also for hospital locations (e.g., big cities vs. rural areas), cleaning efficiency based on different techniques and materials used, cleaning personnel characteristics, HAI and AMR incidence, antibiotic usage, etc. This heterogeneity may have led to possible biases in the results of the budget impact analysis. Future studies involving an extensive set of hospital wards and a mix of hospital settings may be able to provide better estimates of the possible HAIs and related resistances in order to improve the extension of the analysis to the whole hospital admissions. Furthermore, it would be of interest to assess the PCHS antiviral activity, if any, as this action might allow to control virus contamination without worsening the AMR concern of the nosocomial pathogens that are often superinfecting virus-infected hospitalized patients.

Although the conclusion of our study stem from internal medicine wards analysis, we think that it might be extended also to intensive care units, where both HAI prevalence (10%) and AMR of HAI-related microorganisms (32% of total HAIs) are much higher [41], leading to additional savings. Further studies are currently being developed to confirm that generalizability.

## 5. Conclusions

It is known that resistance to a wide range of anti-infective agents today has an increasing impact on global public health and its prevalence is closely related to antibiotic abuse. Antimicrobial agents play a key role in the health sector and increasing rates of antibiotic resistance are a serious threat to infection control, especially in high risk contexts like intensive care units. Excessive prescribing of antimicrobials not only wastes limited health resources, but also creates significant financial burdens and poor health outcomes in different countries.

The European Centre for Disease Prevention and Control (ECDC), the European Medicines Agency (EMA), the European Food and Safety Authority (EFSA), the World Health Organization (WHO) and the European Commission, all recognize that the drivers of AMR are interlinked and lie across the human, animal and environmental health sectors, through a ‘One Health’ perspective. Innovative cleaning systems in healthcare settings have been too long neglected [13] by decision-makers as potential cost-effective approaches to contribute fight HAIs and AMR. The use of sustainable environmental sanitization systems such as the PCHS, might dramatically reduce the HAIs and AMR rates at no additional costs for the healthcare systems. Indeed, the introduction of PCHS as a routine cleaning practice over 5 years might lead to savings ranging from 14 (base case analysis) to 457.5 million euros (acute hospitalizations including insurance reimbursements for severe HAIs, 100% PCHS use).

## Figures and Tables

**Table 1:** Daily costs or minimum-maximum range of antimicrobial drug therapies per patient.

Drug	Intravenous (€)	Inhalation (€)	Oral (€)	Parenteral (€)
Ambisome *	246.71			
Amikacin	1.2–3.06			
Amoxicillin			0.09–0.6	
Amoxicillin/clavulanate	2.58–5.9		0.05–0.33	
Ampicillin	1.96–3.93		0.99	
Ampicillin/sulbactam	4.44–7.86			
Anidulafungin *	354			
Caspofungin *	560.99			
Cefixim			1.14–2.01	
Cefotaxim	1.76			
Ceftazidime	3.37			3.37
Ceftriaxone	0.9–1.32			
Ciprofloxacin	0.11–3.96		0.11–0.88	
Clarithromycin	8.7		0.88	
Clindamicin	1.64		0.84	
Colistin	27			8.95
Daptomicin *	107.2			
Fluconazole	0.54–2		0.99–1.54	
Fosfomycin			1.2–5.68	
Ganciclovir *	13.6			
Gentamicin	0.99–1.24			
Imipenem	9.24			
Imipenem/cilast	9.4–13.58			
Levofloxacin	0.7–0.96		0.2–1.2	
Linezolid	10–151.32			
Meropenem	9.9–13.16			
Metronidazole	0.9–1.8		0.09–0.23	
Nystatin *			0.45	
Oxacillin *	2.99			
Piperacillin/tazobactam	4.43–6.6			
Teicoplanin	5.16–57.75	5.16		36.8
Tigecycline	98.27–107.8			
Trimethoprim/sulfamethoxazole	13.89		0.14–0.22	
Vancomycin	2.5–3.06		3–3.69	
Voriconazole	96.72–96.72		18.84–18.84	

* Drug used to manage healthcare associated infections by a single hospital.

**Table 2:** Populations characteristics before and after matching (PCHS: Probiotic Cleaning Hygiene System, CCC: Conventional Chemical Cleaning).

Populations Characteristics	Unmatched/Matched	PCHS Mean Value	CCC Mean Value	% Bias	% Bias Reduction
Age	U	73.000	71.804	7.4	
	M	73.465	73.465	0	100
Gender (proportion of males)	U	0.471	0.498	−5.5	
	M	0.483	0.483	0	100
Proportion of patients with urinary catheter	U	0.211	0.231	−4.8	
	M	0.200	0.200	0	100
Proportion of patients with self-sufficiency	U	0.649	0.626	4.9	
	M	0.678	0.678	0	100
Proportion of patients with pressure sores	U	0.045	0.064	−8.3	
	M	0.024	0.024	0	100
Proportion of patients with mechanical ventilation	U	0.027	0.039	−6.8	
	M	0.010	0.010	0	100
Proportion of patients with use of antibiotics in the last two weeks	U	0.058	0.091	−12.7	
	M	0.032	0.032	0	100
Proportion of patients with parenteral nutrition	U	0.023	0.036	−7.1	
	M	0.008	0.008	0	100
Proportion of patients with presence at admission of multi-drug resistant organisms	U	0.015	0.022	−5.0	

**Table 3:** Drugs use and treatment durations in the two periods.

Drug	CCC (No HAIs = 191)				PCHS (No HAIs = 100)				PCHS vs. CCC
	Days of Treatment	N. of Treated Pts	Mean Days of Treatment per Pt with HAI	Mean Days of Treatment per Pt with HAI per Specific Drug	Days of Treatment	N. of Treated Pts	Mean Days of Treatment per Pt with HAI	Mean Days of Treatment per Pt with HAI per Specific Drug	Variation in Days of Treatment (%)
Piperacillin/tazobactam	395	45	2.07	8.78	406	39	4.06	10.41	96%
Levofloxacin	269	42	1.41	6.40	15	4	0.15	3.75	−89%
Ciprofloxacin	239	33	1.25	7.24	55	11	0.55	5.00	−56%
Teicoplanin	183	15	0.96	12.20	63	6	0.63	10.50	−34%
Ceftriaxone	176	22	0.92	8.00	90	14	0.90	6.43	−2%
Meropenem	158	14	0.83	11.29	61	6	0.61	10.17	−26%
Vancomycin	133	15	0.70	8.87	38	4	0.38	9.50	−45%
Colistin	79	5	0.41	15.80	40	2	0.40	20.00	−3%
Fluconazole	76	7	0.40	10.86	16	3	0.16	5.33	−60%
Fosfomycin	63	9	0.33	7.00	2	1	0.02	2.00	−94%
Anidulafungin	51	5	0.27	10.20	0	0	0.00	0.00	−100%
Metronidazole	45	7	0.24	6.43	25	3	0.25	8.33	6%
Ampicillin/sulbactam	40	5	0.21	8.00	8	1	0.08	8.00	−62%
Amoxicillin/clavulanate	34	5	0.18	6.80	104	11	1.04	9.45	484%
Imipenem	30	2	0.16	15.00	26	3	0.26	8.67	66%
Linezolid	30	2	0.16	15.00	58	4	0.58	14.50	269%
Tigecycline	28	3	0.15	9.33	18	2	0.18	9.00	23%
Cefixim	23	1	0.12	23.00	3	1	0.03	3.00	−75%
Trimethoprim/sulfamethoxazole	22	3	0.12	7.33	0	0	0.00	0.00	−100%
Ambisome	21	1	0.11	21.00	0	0	0.00	0.00	−100%
Gentamicin	20	2	0.10	10.00	2	1	0.02	2.00	−81%
Voriconazole	19	3	0.10	6.33	0	0	0.00	0.00	−100%
Ampicillin	18	3	0.09	6.00	0	0	0.00	0.00	−100%
Imipenem/cilast	16	2	0.08	8.00	8	1	0.08	8.00	−5%
Amikacin	15	2	0.08	7.50	25	1	0.25	25.00	218%
Caspofungin	13	1	0.07	13.00	0	0	0.00	0.00	−100%
Ganciclovir	12	1	0.06	12.00	0	0	0.00	0.00	−100%
Clarithromycin	11	3	0.06	3.67	14	2	0.14	7.00	143%
Penicillin	10	1	0.05	10.00	0	0	0.00	0.00	−100%
Daptomicin	9	2	0.05	4.50	0	0	0.00	0.00	−100%
Amoxicillin	8	2	0.04	4.00	7	2	0.07	3.50	67%
Nystatin	7	1	0.04	7.00	0	0	0.00	0.00	−100%
Ceftazidime	6	1	0.03	6.00	0	0	0.00	0.00	−100%
Clindamicin	5	1	0.03	5.00	0	0	0.00	0.00	−100%
Cefotaxim	0	0	0.00	0.00	11	1	0.11	11.00	100%
Oxacillin	0	0	0.00	0.00	9	1	0.09	9.00	100%
Total	2264				1104				

## Appendix A

Cost comparison between Conventional Chemical Cleaning (CCC) and PCHS (CONSIP and AFIDAMP specification [48,49]).

**Table A1:** Labor Analysis—Labor Value Table of the Italian Ministry of Labor.

Level	€/Hour	Employment	Value €/Hour
2° level	€15.88	85.00%	€13.50
3° level	€16.65	12.00%	€2.00
4° level	€17.52	3.00%	€0.53
TOTAL			€16.02
	**Item**	**Cost component**	**VALUE €/HOUR**
Analysis of “material” needs			
	Machinery	1.40%	€0.22
	Equipment	1.80%	€0.29
	Cleaning products	2.50%	€0.40
	Consumables	2.00%	€0.32
	Treasury	4.00%	€0.64
Analysis of “ancillary” needs			
	Overheads	7.00%	€1.12
	Profit	3.00%	€0.48
		**€/hour GROSS REVENUE**	€19.50

**Table A2:** From Annex 5—Technical specification.

Area Code	Description	Locations Included
MR1	Ward with hospitalization—Medium risk	Inpatient rooms, medication room, ward depot, nurses’ work room, herbal tea room, ward kitchen, dining room, related to the following departments: First aid or ER, day hospital, and other departments not included in the homogeneous area AR0
MR3	Corridors and waiting rooms—Medium risk	Corridors, waiting rooms inside the wards and medium-risk diagnosis areas, lifts for patient transport, living rooms inside the wards
MR4	Hospital toilets, staff toilets or in any case open 7 days a week	Toilets inside the wards, operating departments and diagnostic areas, (regardless of the risk area they belong to) drains, and other similar rooms open 7 days a week

**Table A3:** Specification of activities and time spent (hours)—MR1—Example based on hospital stay room with 2 beds (m^2^ 25).

Activity Description		Frequency	CCC Time Spent	PCHS Time Spent
**Emptying of waste bins with closure and transport to the solid or similar waste collection point and replacement of the bag and/or container**		*2 per day*	0.025	0.025
Wet dusting and cleaning including the removal of stains on horizontal and vertical surfaces up to 180 cm, on furnishings, sanitary facilities (beds, bedside tables, bed headboards, dividing curtains, IV stands including wheels, serving tables, trolleys, stretchers, prams, walkers, supports, etc.) and common contact points (telephones, switches and push-button panels, audiovisual equipment, handles, handrails, etc.) light points, fan coils, internal windowsills, doors, windows and other washable surfaces		*daily*	0.1917	0.1917
Wet sweeping with removal of dust and waste from all floors, with disposable and/or suction gauze.		*daily*	0.0208	*
Floor washing, after moving the easily removable furnishings. Reposition on dry floor of what was previously moved. If using a washer dryer, finish with manual cleaning the points not reached by washing by machine.		*daily*	0.05	0.05
Cleansing and disinfection of waste bins, bag carriages and waste bins.		*weekly*	0.0143	0.0143
Maintenance of protective floor treatments (spray cleaning or spray buffing).		*monthly*	0.0033	0.0033
Debinding with total or partial removal of the film and subsequent waxing of the floors.	*de-waxing*	*every six months*	0.0046	0.0046
	*waxing*		0.0009	0.0009
Cleaning the external side of the fixtures, including glass surfaces, bins and external window sills if accessible from the inside in compliance with safety regulations.		*every three months*	0.0003	0.0003
**Total working hours CONTINUOUS ACTIVITIES on a day basis (7/7)**			0.2875	0.2667
**Total working hours PERIODIC ACTIVITIES (from weekly to annual) on a day basis (7/7)**			0.0234	0.0234
**TOTAL DAILY HOURS**			0.3109	0.29

* Operation that is not provided (or provided partially) as it is overcome by the use of PCHS, which involves the use of a cloth that combines dusting and washing activities.

**Table A4:** Specification of activities and time spent (hours)—MR3—Example on a section of ward corridor (m^2^ 30).

Activity Description		Frequency	CCC Time Spent	PCHS Time Spent
**Emptying of waste bins with closure and transport to the solid or similar waste collection point and replacement of the bag and/or container**		*2 per day*	0.0083	0.0083
Wet dusting and cleaning including the removal of stains of horizontal and vertical surfaces up to 180 cm, on furniture, and common contact points (telephones switches and push-button panels, handles, handrails, etc.), fan coils, window sills, doors, internal parts of the fixtures including glass surfaces, and other washable surfaces.		*2 per day*	0.0333	0.0333
Wet sweeping with removal of dust and waste from all floors, with disposable and/or suction gauze.		*2 per day*	0.0376	0.0188 *
Floor washing, after moving the easily removable furnishings. Reposition on dry floor of what was previously moved. If using a washer dryer, finish with manual cleaning the points not reached by washing by machine.		*daily*	0.05	0.05
Cleansing and disinfection of waste bins, bag carriages and waste bins.		*weekly*	0.0048	0.0048
Maintenance of protective floor treatments (spray cleaning or spray buffing).		*monthly*	0.004	0.004
Debinding with total or partial removal of the film and subsequent waxing of the floors.	*de-waxing*	*every six months*	0.0055	0.0055
	*waxing*		0.0011	0.0011
Cleaning the external side of the fixtures, including glass surfaces, bins and external window sills if accessible from the inside in compliance with safety regulations.		*every three months*	0.0003	0.0003
**Total working hours CONTINUOUS ACTIVITIES on a day basis (7/7)**			0.1292	0.1104
**Total working hours PERIODIC ACTIVITIES (from weekly to annual) on a day basis (7/7)**			0.0156	0.0156
**TOTAL DAILY HOURS**			0.1448	0.126

* Operation that is not provided (or provided partially) as it is overcome by the use of PCHS, which involves the use of a cloth that combines dusting and washing activities.

**Table A5:** Specification of activities and time spent (hours)—MR4—Example of one toilet service with a cabinet, a bidet, a shower tray, a sink, a mirror, a shelf (m^2^ 4).

Activity Description	Frequency	CCC Time Spent	CCC Time Spent
Emptying of waste bins with closure and transport to the solid or similar waste collection point and replacement of the bag and/or container	*2 per day*	0.0167	0.0167
Cleaning and disinfection of sanitary fixtures, showers, shower cubicles, washable walls and all the accessories and sanitary furnishings present in these areas (including pipe cleaners and brush holders).	*2 per day*	0.3	0.3
		0.15	0.15
Cleaning of soap dispensers, wipes, toilet paper.	*2 per day*	0.15	0.15
		0.075	0.075
Wet dusting with the help of specific products of horizontal surfaces and up to 180 cm, radiators, fan coils, internal windowsills, push-button panels, handles, crystals, mirrors, shelves, furnishings, etc.	*2 per day*	0.1833	0.1833
Wet rubbish with removal of dust and waste from the floors.	*2 per day*	0.0033	*
		0.0033	*
Floor washing and disinfection.	*2 per day*	0.008	0.008
		0.008	0.008
Descaling of sanitary ware, taps and neighboring areas	*2 per week*	0.0714	0.0714
Spiderweb removals	*weekly*	0.001	0.001
Cleaning of rubbish bins, bag holder trolleys for laundry	*weekly*	0.0095	0.0095
Thorough cleaning of floors, if necessary carry out descaling. Previously transport any furniture and furnishings, after thorough cleaning, outside the room.	*monthly*	0.0012	0.0012
Wet dusting of convector heaters, heaters, air conditioning appliances, air vents, bins.	*monthly*	0.0066	0.0066
Mechanical aspiration of all areas not manually accessible beyond cm. 180 including lighting fixtures, convectors, heaters, conduits, bins, air vents, etc.	*every four months*	0.0005	0.0005
Cleaning the external side of the fixtures, including glass surfaces, bins and external window sills if accessible from the inside in compliance with safety regulations	*every three months*	0.0002	0.0002
**Total working hours CONTINUOUS ACTIVITIES on a day basis (7/7)**		0.9691	0.9624
**Total working hours PERIODIC ACTIVITIES (from weekly to annual) on a day basis (7/7)**		0.019	0.019
**TOTAL DAILY HOURS**		0.9881	0.9815

* Operation that is not provided (or provided partially) as it is overcome by the use of PCHS, which involves the use of a cloth that combines dusting and washing activities.

**Table A6:** Cost calculation considering area codes MR1, MR3 and MR4.

Description	CCC		PCHS
**Total working hours CONTINUOUS ACTIVITIES on a day basis (7/7) (MR1+MR3+MR4)**	1.3858	1.3395	
Total working hours PERIODIC ACTIVITIES (from weekly to annual) on a day basis (7/7) (MR1+MR3+MR4)	0.058	0.058	
TOTAL DAILY HOURS	1.4438	1.3975	
HOURLY COST	€19.50	€19.50	
MONETIZATION DAY SERVICE	€28.15	€27.25	
SAVINGS			€0.90 (=3.20%)
